# Exosomal ROR1 in peritoneal fluid identifies peritoneal disseminated PDAC and is associated with poor survival

**DOI:** 10.3389/fimmu.2024.1253072

**Published:** 2024-05-23

**Authors:** Anke Mittelstädt, Anna Anthuber, Paul David, Malgorzata Podolska, Alan Bénard, Maximilian Brunner, Christian Krautz, Anne Jacobsen, Axel Denz, Klaus Weber, Susanne Merkel, Danilo Hackner, Timur Buniatov, Lotta Roßdeutsch, Bettina Klösch, Izabella Swierzy, Frederik J. Hansen, Deike Strobel, Yurdagül Zopf, Jan-Ole Baur, Jan Van Deun, Carol Immanuel Geppert, Andreas Gießl, Sebastian Lettmaier, Sabine Semrau, Robert Grützmann, Dina Kouhestani, Georg F. Weber

**Affiliations:** ^1^ Department of Surgery, University Hospital Erlangen, Erlangen, Germany; ^2^ Department of Gastroenterology, University Hospital Erlangen, Erlangen, Germany; ^3^ Department of Internal Medicine 5, University Hospital Erlangen, Erlangen, Germany; ^4^ Department of Dermatology, University Hospital Erlangen, Erlangen, Germany; ^5^ Department of Pathology, University Hospital Erlangen, Erlangen, Germany; ^6^ Department of Ophthalmology, University Hospital Erlangen, Erlangen, Germany; ^7^ Department of Radiation Oncology, University Hospital Erlangen, Erlangen, Germany; ^8^ Deutsches Zentrum für Immuntherapie (DZI), Friedrich Alexander University Erlangen-Nuremberg and Universitätsklinikum Erlangen, Erlangen, Germany; ^9^ Comprehensive Cancer Center, Friedrich-Alexander-Universität Erlangen-Nürnberg, Universitätsklinikum Erlangen, Erlangen, Germany

**Keywords:** ROR1, exosomes, peritoneal lavage, peritoneal fluid, PDAC, peritoneal carcinomatosis, targeted therapy, biomarker

## Abstract

**Background:**

Pancreatic ductal adenocarcinoma (PDAC) is one of the deadliest forms of cancer and peritoneal dissemination is one major cause for this poor prognosis. Exosomes have emerged as promising biomarkers for gastrointestinal cancers and can be found in all kinds of bodily fluids, also in peritoneal fluid (PF). This is a unique sample due to its closeness to gastrointestinal malignancies. The receptor tyrosine kinase-like orphan receptor 1 (ROR1) has been identified as a potential biomarker in human cancers and represents a promising target for an immunotherapy approach, which could be considered for future treatment strategies. Here we prospectively analyzed the exosomal surface protein ROR1 (exo-ROR1) in PF in localized PDAC patients (PER-) on the one hand and peritoneal disseminated tumor stages (PER+) on the other hand followed by the correlation of exo-ROR1 with clinical-pathological parameters.

**Methods:**

Exosomes were isolated from PF and plasma samples of non-cancerous (NC) (n = 15), chronic pancreatitis (CP) (n = 4), localized PDAC (PER-) (n = 18) and peritoneal disseminated PDAC (PER+) (n = 9) patients and the surface protein ROR1 was detected via FACS analysis. Additionally, soluble ROR1 in PF was analyzed. ROR1 expression in tissue was investigated using western blots (WB), qPCR, and immunohistochemistry (IHC). Exosome isolation was proven by Nano Tracking Analysis (NTA), WB, Transmission electron microscopy (TEM), and BCA protein assay. The results were correlated with clinical data and survival analysis was performed.

**Results:**

PDAC (PER+) patients have the highest exo-ROR1 values in PF and can be discriminated from NC (p <0.0001), PDAC (PER-) (p <0.0001), and CP (p = 0.0112). PDAC (PER-) can be discriminated from NC (p = 0.0003). In plasma, exo-ROR1 is not able to distinguish between the groups. While there is no expression of ROR1 in the exocrine pancreatic tissue, PDAC and peritoneal metastasis show expression of ROR1. High exo-ROR1 expression in PF is associated with lower overall survival (p = 0.0482).

**Conclusion:**

With exo-ROR1 in PF we found a promising diagnostic and prognostic biomarker possibly discriminating between NC, PDAC (PER-) and PDAC (PER+) and might shed light on future diagnostic and therapeutic concepts in PDAC.

## Introduction

Pancreatic ductal adenocarcinoma (PDAC) is one of the deadliest forms of cancer, with an overall five-year survival rate of less than 10% in the US (1). Despite improved diagnostic methods, multimodal therapy concepts and surgical techniques, the 5-year survival rate even after radical surgery with adjuvant therapy in localized tumor stages remains 30–40% (2, 3). Early detection and effective treatment are critical for improving patient outcomes. Consequently, in recent years, there has been growing interest in finding biomarkers for the early detection and monitoring of PDAC. Peritoneal relapse occurs in approximately 30% of the relapse patterns and is hence a major cause for this poor prognosis (4, 5). Therefore, it is important to find prognostic biomarkers and to develop treatment strategies that consider the high risk of peritoneal relapse. Peritoneal lavage fluid and ascites (together referred to as peritoneal fluid (PF)) are unique samples due to their closeness to gastrointestinal malignancies and are already in use especially for cytological analyses (6).

Exosomes, small extracellular vesicles (30–150 nm) released by all kinds of cells and also cancer cells, have emerged as promising biomarkers for gastrointestinal cancers due to their ability to carry specific cargo, including proteins and nucleic acids. They can be found in all kinds of bodily fluids (e.g. blood, saliva, urine, ascites) which makes them even more interesting (7, 8).

Glypican-1 positive exosomes in serum are able to distinguish benign pancreatic disease from early- and late-stage pancreatic cancer and were also correlated with tumor burden and survival (8).

The receptor tyrosine kinase-like orphan receptor 1 (ROR1) plays an essential role in embryogenesis and is overexpressed in many types of malignant tumors. Contrarily, ROR1 is mostly absent in normal human tissues. However, it can be found for example in the parathyroid gland as well as in the pancreatic islet cells (9). Studies have demonstrated that ROR1 plays an important role in oncogenesis by activating cell survival signaling events, particularly the non-canonical WNT signaling pathway. The function as a tyrosine kinase is still poorly understood (10).

ROR1 can be found on the surface of exosomes and has been identified as a potential biomarker in human ovarian cancer (11), lung cancer (12), but also in PDAC (13, 14). The depletion of ROR1 in PDAC suppresses tumor growth, recurrence after chemotherapy, and metastasis (15). Additionally, ROR1 represents a promising target for an immunotherapy approach (10, 16) which could be considered for future treatment strategies.

Here we prospectively analyzed the exosomal surface protein ROR1 (exo-ROR1) in PF in localized, locally advanced and oligo-metastasized PDAC patients (PER-) on the one hand and peritoneal disseminated tumor stages (PER+) on the other hand followed by the correlation of exo-ROR1 with clinical-pathological parameters.

## Materials and methods

### Patients

The study includes samples of 46 patients with localized, locally advanced, or oligo metastasized PDAC (PDAC (PER-)) (n = 18), PDAC with peritoneal carcinomatosis (PDAC (PER+)) (n = 9), chronic pancreatitis (CP) (n = 4), and non-cancerous controls (NC) (n = 15), who received surgery or ascites puncture at the University Hospital of Erlangen between 2021 and 2023. The patients with PDAC (PER-) received laparotomy in curative intention. 4 PDAC (PER-) patients were staged as M1. Three of these patients had a small liver metastasis found after laparotomy and one patient had a positive interaortocaval lymph node after histopathological examination. We categorized the localized, locally advanced or oligo metastasized PDAC patients as PDAC (PER-). PDAC (PER+) patients were operated due to ileus or even in curative intention before incidental intraoperative finding of peritoneal carcinomatosis. Others had a relapse with ascites and radiologic signs or known peritoneal carcinomatosis from earlier surgery and received ascites puncture. Patients with CP received surgery for removing the pancreatic head due to congested pancreatic duct. The NC patients received elective open or laparoscopic surgery, mostly due to hernias or uncomplicated symptomatic cholecystolithiasis.

All patients were eligible for inclusion and signed informed consent prior to medical intervention. The study was approved by the ethical committee of Erlangen (UKER 180_19 B).

Follow-up data were collected either through follow-up visits at the university hospital or through written correspondence with the patients’ treating physicians.

All data was collected prospectively.

### Sample collection

#### Peritoneal lavage fluid

Directly after opening the abdominal cavity (open surgery as well as laparoscopic surgery), 100 ml physiological saline solution were used for a peritoneal lavage. Subsequently as much fluid as possible (but at least 50 ml) was recollected, centrifuged at 350 g for 5 minutes to remove cells and cell debris and stored at -80°C.

#### Ascites

Patients with ascites and known peritoneal carcinomatosis of PDAC received ascites puncture to collect at least 200 ml ascites. In some cases, ascites was collected intraoperatively. 100 ml ascites were sent to pathology for cytology tests. The other 100 ml were centrifuged at 350 g for 5 minutes to remove cells and cell debris and stored at -80°C.

#### Blood samples

Peripheral blood (14 ml) was collected in an ethylene diaminetetraacetic acid (EDTA) coated tube prior to surgery. Plasma samples were separated from peripheral blood, centrifuged at 350 g for 10 minutes with brake 4 to remove dead cells and cell debris and stored at -80°C until subsequent analysis.

#### Tissue

Tumor and normal pancreatic tissues of PDAC and pancreatitis patients were obtained after surgery. Fresh tissue was transferred to pathology and part of it fixed in formaldehyde for further processing and the other part stored at -80°C.

### Exosome isolation

**Figure 1:**
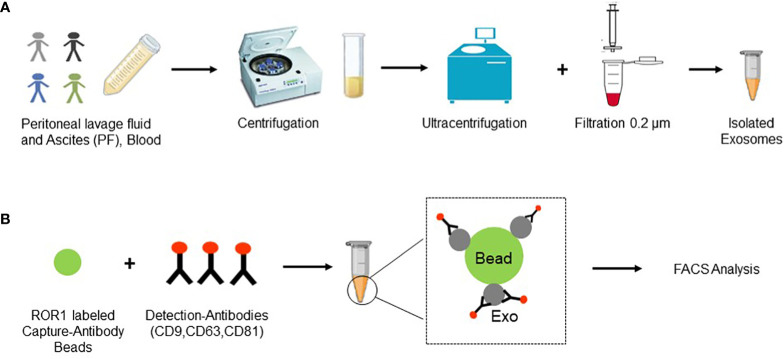
Experimental layout. **(A)** Samples (PF and blood) were collected and several centrifugation steps and two ultracentrifugation steps were performed. Additionally, samples were filtered through a 0.2 µm filter and finally exosomes were collected in 500 µl of PBS. **(B)** Exosomes were incubated with ROR1 labeled capture antibody beads and following with detection antibodies for the three known exosomal markers CD9, CD63, and CD81. Finally, FACS analysis was performed. Created with BioRender.com.

Exosome isolation was performed through consequent centrifugation steps: plasma samples (4.8 ml) and PF samples (30–50 ml) were centrifuged at 300 g for 10 minutes (removal of cellular components), 2000 g for 30 minutes (removal of cellular debris), 10,000 g for 45 minutes (removal of bigger extracellular vesicles (EVs)). For the enrichment of exosomes two ultracentrifuge steps at 100,000 g for 2 hours were performed. Pellets with the EVs were resuspended in 3 ml PBS and filtrated through Millex-GV Filters, a 25 mm sterile syringe filter with a 0.22 µm pore size Polyethersulfone membrane (Catalog No. SLMP025SS, Merck, Darmstadt, Germany) in between the two ultracentrifugation steps. EVs pellets were resuspended in 500 µl of PBS (**Figure 1A**).

### BCA protein assay

The EV and respective exosome concentration was measured by Pierce BCA protein assay Kit (Catalog No. 23227, Thermo scientific) using bovine serum albumin (BSA) standards. The kit was used according to manufacturer’s recommendations. For data analysis the concentrations of the isolated exosomes from peritoneal lavage samples were multiplied by factor 4. Physiological peritoneal fluid volume is somewhere between 30 and 50 ml (17, 18). Therefore, we defined an approximate volume of 33 ml. Adding 100 ml of saline solution resulted in a 1:4 dilution, which was considered for calculation of the final concentration. Concentrations of ascites samples were not changed.

### Nanoparticle tracking analysis

Particle size distribution in the purified EVs was determined by using Zetaview PMX-110 (Particle Metrics, Inning am Ammersee, Germany), which is equipped with a 405 nm laser. This NTA instrument was also used to measure the particle concentrations. Before sample measurement, the instrument was calibrated using 100 nm polystyrene beads diluted in water according to manufacturer’s instructions. Cell temperature was maintained at 25°C for all measurements. Samples were diluted to an appropriate concentration in PBS, in a total volume of 1 ml. Eleven cell positions were scanned for each measurement cycle, with video recorded at 30 frames per second. Additional capture settings were: gain 719.52, shutter 50, minimum trace length 15. ZetaView software version 8.05.12 was used to analyze the recorded videos with the following settings: minimum brightness 25, maximum brightness 255, minimum area 5, and maximum area 200. Finally, the EVs concentration was calculated using the appropriate dilution factors according to the manufacturer’s recommendation.

### Immunogold labelling and electron microscopy

Fixed EV specimens (4% PFA in PBS mixed 1:1 with EV) were placed onto 10 min UV irradiated 300-mesh formvar/carbon coated grids and allowed to absorb to the formvar for 5 min. For immunogold staining the grids were placed into 20 µl 0.01% Tween/PBS (10 min) and after that into a blocking buffer (0.5% fish gelatin with 0.1% ovalbumin in PBS) for a block step for 1 h. Without rinsing, the grids were immediately placed into the primary antibody (diluted in blocking buffer) at the appropriate dilution overnight at 4°C (1:100 anti-CD9 Abcam, ab236630). As controls, some of the grids were not exposed to the primary antibody. The next day, all the grids were rinsed with PBS then floated on drops of the appropriate secondary antibody attached with 10-nm gold particles (AURION 1:30) for 2 h at room temperature. Grids were rinsed 3 times with PBS and were placed in 1% glutaraldehyde (in PBS) for 5 min. After rinsing in PBS and distilled water, the grids were stained for contrast using 2% uranyl oxalate solution (pH7 for 5 min in dark). Afterwards the grids were incubated in drops of methyl cellulose-uranyl oxalate (8 parts 2% methyl cellulose, 1 part ddH_2_O, 1 part 4% uranyl acetate (in water), pH4, sterile filter) for 10 min on ice (dark) according to Théry et al. (19). Next, grids were removed with stainless steel loops and excess fluid was blotted by gently pushing on Whatman filter paper. After air-drying, the samples were examined and photographed with a Zeiss EM10 electron microscope (Zeiss, Jena, Germany) and a Gatan SC1000 Orius™ CCD camera (GATAN, Munich, Germany) in combination with the DigitalMicrograph™ software (GATAN, Pleasanton, CA, USA). Images were adjusted for contrast and brightness using Adobe Photoshop CC 2018 (Adobe Systems, San José, CA, USA).

### MACSPlex exosome assay and flow cytometry analysis

The screening assay (Catalog No.130–108-813, MACSPlex Human Exosome Kit; Miltenyi, Bergisch Gladbach, Germany) was previously described (20, 21). In brief, the assay is based on 4–8 µm diameter poly-styrene beads, labelled with different amounts of 2 dyes (phycoerythrin and fluorescein isothiocyanate) to generate 39 different bead subsets subjected to flow cytometry analysis. Each bead subset is conjugated with a different capture antibody that recognizes EVs carrying the respective antigen (37 EV surface epitopes - including ROR1 - plus 2 isotype controls). Beads were incubated with the samples overnight. On the next day EVs bound to beads were detected by allophycocyanin-conjugated anti-CD9, anti-CD63, and anti-CD81 antibodies (**Figure 1B**). Finally, samples were analyzed with the BD LSR Fortessa ™ (BD, New Jersey, USA) special order research product (with blue, red, violet, UV, YellGrn laser). PBS was used to measure background signal. Median fluorescence intensity (MFI) of each EV marker was normalized to the mean MFI for specific EV markers (CD9, CD63, and CD81). For calculation of relative number of exosome surface markers, first the median signal intensity of each bead obtained from the buffer as control sample was subtracted from the signal intensities of the respective beads incubated with sample. Finally, the signal intensities of all beads were divided by normalization factor of the respective sample.

### ELISA of ROR1

ELISA of ROR1 in PF samples was performed with Human ROR1 ELISA Kit Cat. No. EH395RB (Invitrogen, ThermoFisher Scientific, Massachusetts, USA) according to manufactures instructions. In brief, 100 µl of standards and samples were added to the 96 well plate. After incubation time and washing biotin-conjugated detection antibody was added. Subsequently to incubation and washing, Streptavidin-HRP solution was added to the wells. Following incubation and washing TMB Substrate was added and the reaction was stopped 30 min later. The absorbance was read at 450 nm in the plate reader (SpectraMax M3 Multi-Mode Microplate Reader, Molecular Devises, San Jose, CA, USA). For data analysis the concentrations of the peritoneal lavage samples were multiplied by factor 4 in the same way as described in the BCA protein assay above.

### Protein extraction from tissue

Tissues were lysed in RIPA buffer (Cat. #89900, Thermo Fisher Scientific) containing protease and phosphatase inhibitor (Cat. #78442, Thermo Fisher Scientific) as well as metal beads. Lysing was performed with TissueLyser II machine (Qiagen, Venlo, Netherlands) at a frequency of 30/s for 4 minutes. Afterwards samples were centrifuged at 10,000 g and supernatant was frozen at -20°C degrees until further use.

### Western blot

The protein concentration of lysed tissue and exosomes were determined photometrically using a BCA Protein Assay Kit (Catalog No. 23227, Thermo Fisher Scientific). Equal amounts of total protein (10 µg) were separated on 4–12% NUPAGE Bis–Tris gels (Cat. #NP0322BOX; Thermo Fisher Scientific) using the Mini Gel Tank chamber system (Cat. #A25977, Invitrogen), and proteins were transferred to a nitrocellulose membrane (Cat. #GE10600003, Sigma Aldrich). Membranes were then blocked in blocking buffer (Cat. #A0830.1000, AppliChem GmbH) for 1 h at room temperature and incubated with ROR1 (Cat. #16540, Cell Signaling) CD81 (Cat. #56039, Cell Signaling) and β-Actin (Cat. #4970, Cell Signaling) overnight at 4°C. HRP-linked anti-rabbit IgG (Cat. #7074, Cell Signaling) were used as the secondary antibodies. Signal detection was performed using an Amersham Imager 600 (Pittsburgh, PA, USA) with SignalFire™ ECL Reagent (Cat. #6883S, Cell Signaling).

### Quantitative polymerase chain reaction

The RNA extraction from whole tissue was conducted using the RNeasy mini kit (Qiagen, Venlo, Netherlands). Subsequently, 1 µg of total RNA was reverse transcribed into complementary DNA using Moloney murine leukemia virus reverse transcriptase (Thermo Fisher Scientific) with random hexamer oligonucleotides as primers (Thermo Fisher Scientific). Amplification was carried out using the Biorad CFX-Connect Real-time-System and the SYBR Green (Eurogentec, Seraing, Belgium) detection system. Data analysis was performed using Bio-Rad CFX Manager 3.1 software. The mRNA content for ROR1 was normalized to MLN51 mRNA levels for human genes. Gene expression quantification was done using the ΔΔCt method, where the expression level was arbitrarily set to 1 for a sample from the control group, and values for other samples were calculated relative to this reference. The primer sequences for the quantified genes are as follows: MLN51 forward: 5´-TAA TCC CAG TTA CCC TTA TGC TCC A-3´, MLN51 reverse: 5´- GTT ATA GTA GGT CAC TCC TCC ATA TAC CTG T-3´; ROR1 forward: 5´-TTC TTC ATT TGC GTC TGT CG-3´, ROR1 reverse: 5´-GGC ACA CTC ACC CAA TTC TT-3´.

### Histology and immunostaining

The resected tissues were promptly fixed in 4% paraformaldehyde (Nakalai Tesque, 09154–56) at 4°C for 16 hours. Following fixation, the samples were embedded in paraffin and sliced into 4-μm sections for histological examination. Hematoxylin and eosin (H&E) staining were performed using standard protocols. For immunohistochemistry (IHC), antigen retrieval was conducted at 120°C for 1 min utilizing 0.01 M citrate buffer (pH 7.0). Subsequently, sections were treated with 3% H2O2 to quench endogenous peroxidase activity, followed by blocking of nonspecific binding with Tris-buffered saline/0.1% Tween-20 (TBS-T) containing 5% goat serum (Jackson Immuno Research Laboratories, 005–000-001). Primary antibody was rabbit anti-ROR1 (Thermo Fisher Scientific, PA5–50830; 1:500). Secondary antibodies comprised biotinylated Goat Anti-Rabbit IgG antibody (Sigma-Aldrich 21537), Avidin-Biotin-Complex ABC, detected using a Liquid DAB+ Substrate Chromogen System (DAKO, K3468). Imaging was performed using a Leica DM4000 B microscope (Leica, Wetzlar, Germany).

### Statistical analysis

Statistical analysis was performed using GraphPad Prism version 9 and IBM SPSS version 28. Variable distribution was identified by Shapiro-Wilk test and Kolmogorov-Smirnov test. Nominal and ordinal data was analyzed by Pearson’s chi-squared test, metric normally distributed data by ANOVA (or t-test in between two groups) and non-normally metric data by Kruskal-Wallis test. Column analysis for non-normally distributed data was done by Mann-Whitney U test. In ROC-curve analysis the estimated cut-off values with the correspondent sensitivity and specificity of GraphPad Prism version 9 were used. For survival analysis we divided the cancer patients in low and high expression of exo-ROR1 according to the median of all exo-ROR1 values. Survival data was analyzed by Kaplan-Meier method and log rank test. Statistical significance was set at p ≤ 0.05.

## Results

### Clinical parameters show the severe illness of the PDAC (PER+) patients

The baseline characteristics of the clinical parameters (**Table 1**) show some significant differences between the subgroups. The CP patients are younger than the cancer patients (median pancreatitis 48.5 years vs. PDAC (PER-) 68.5 years (p = 0.05) or PDAC (PER+) 69 years (p = 0.001)). Also, the NC patients are slightly younger than the cancer patients (median NC 62 years vs. median PDAC (PER-) 68.5 years or PDAC (PER+) 69 years), but not statistically significant. The overall significance regarding age in between the groups equals p = 0.003.

**Table 1:** Baseline characteristics of all included patients stratified into the subgroups Non Cancer (n=15), Pancreatitis (n=4), PDAC (PER-) (n=18) and PDAC (PER+) (n=9).

	All patients	Subgroups				
		Non Cancer	Pancreatitis	PDAC (PER-)	PDAC (PER+)	p
**N (%)**	46 (100)	15 (32.6)	4 (8.7)	18 (39.1)	9 (19.6)	–
**Age (years), median (IQR)**	67 (22)	62 (23)	48.5 (23)	68.5 (23)	69 (10)	**0.003**
**Sex, n (%)**						0.634
** Female**	23 (50)	6 (40)	3 (75)	9 (50)	5 (55.6)	
** Male**	23 (50)	9 (60)	1 (25)	9 (50)	4 (44.4)	
BMI (kg/m^2^) (n=44)*, median (IQR)	24.85 (5.6)	28 (6.3)	24.4 (3.2)	24 (4.2)	22.7 (7.1)	0.154
**ASA, n (%)**						**0.013**
** I**	1 (2.2)	1 (6.7)	0 (0)	0 (0)	0 (0)	
** II**	24 (52.2)	11 (73.3)	1 (25)	12 (66.7)	0 (0)	
** III**	20 (43.5)	3 (20)	3 (75)	6 (33.3)	8 (88.9)	
** IV**	1 (2.2)	0 (0)	0 (0)	0 (0)	1 (11.1)	
**Diabetes, n (%)**	9 (19.6)	2 (13.3)	0 (0)	5 (27.8)	2 (22.2)	0.541
Preoperative blood results, median (IQR)						
WBC (x10^3^/µl) (n=42)*	6.2 (3.8)	6.5 (2.8)	6.1 (2.2)	5.9 (4.1)	6.0 (11.5)	0.877
Hemoglobin (g/dl) (n=42)*	13.5 (2.6)	14.2 (1.9)	13.7 (4.6)	12.6 (2.8)	10.8 (4.1)	**0.021**
CRP (mg/l) (n=43)*	3.1 (14.1)	2.1 (2)	3.4 (6.2)	4.5 (20)	17.4 (59.8)	**0.010**
Lipase (U/l) (n=39)*	33 (18)	33 (18)	11.9 (123.85)	29 (48)	5 (15.1)	**0.022**
Creatinine (mg/dl) (n=45)*	0.88 (0.38)	0.93 (0.4)	0.72 (0.5)	0.81 (0.4)	0.92 (0.5)	0.495
Albumin (g/l) (n=28)*	39.8 (10.5)	–	41 (5.15)	39.6 (9.1)	33 (15.2)	0.147
Bilirubin (mg/dl) (n=42)*	0.7 (0.8)	0.5 (0.3)	0.45 (0.3)	1.0 (1.2)	0.7 (0.8)	**0.008**
ɤGT (U/l) (n=38)*	73 (120)	18 (23)	32.5 (368.5)	111 (123.3)	112 (117)	**0.020**
Quick (%) (n=40)*	86.5 (28.5)	91.5 (26)	86.5 (23.8)	94 (18.8)	66.5 (24.3)	**0.041**
Preoperative tumor markers, median (IQR)						
CEA (ng/ml) (n=22)*	3.8 (7.3)	–	2.9	3.1 (7.5)	5 (12)	0.515
CA19–9 (U/ml) (n=21)*	120 (1837.2)	–	8.0	115.1 (2517.6)	827 (6489)	0.253

*missing data, WBC, white blood cell count; **ɤ**GT, gamma-glutamyl transferase. Bold: significant values.

The groups also differ in the ASA Score (p = 0.013). The NC patients have lower ASA Scores than the cancer patients. In the PDAC (PER+) group most patients have an ASA Score of 3 (88.9%) whereas in the PDAC (PER-) group most patients have an ASA Score of 2 (66.7%).

Regarding preoperative blood results the cancer patients and especially PDAC (PER+) show lower hemoglobin levels (PDAC (PER+) 10.8 g/dl vs. NC 14.2 g/dl) (p = 0.021), higher CRP (p = 0.01), bilirubin (p = 0.008) and gamma-glutamyl transferase (**ɤ**GT) levels (p = 0.02). The quick value is lower in the PDAC (PER+) group in comparison to the other groups (PDAC (PER+) 66.5% vs. NC 91.5%, CP 86.4%, PDAC (PER-) 94%; p = 0.041). There are also statistically significant differences of the lipase levels whereas CP has the highest levels with a median of 11.9 U/l, IQR (123.85) (p = 0.022).

No significant differences can be detected in gender, BMI, existing Diabetes, white blood cell count (WBC), creatinine, albumin and the tumor markers CEA und CA19–9.

Concerning the tumor characteristics (**Table 2**) more PDAC (PER+) patients had a preoperative or preinterventional systemic therapy (78% vs. 11% for PDAC (PER-), p<0.001). The PDAC (PER+) patients had also a higher UICC tumor stage (p = 0.002), higher R status (p<0.001) and a higher metastasis rate (p<0.001). There were no differences regarding tumor size, invasion into the lymph nodes, perineural invasion and grading.

**Table 2:** Tumor characteristics of cancer patients stratified in the two groups PDAC (PER-) (n=18) and PDAC (PER+) (n=9).

	All PDAC n (%)	PDAC (PER-) n (%)	PDAC (PER+) n (%)	p
**All patients (n=27)**	27 (100)	18 (67)	9 (33)	**-**
**c/pM1**	13 (48.1)	4 (22)	9 (100)	**<0.001**
**UICC tumor stage**				**0.002**
** c/p Stage I**	2 (7.4)	2 (11)	–	
** c/p Stage II**	8 (29.6)	8 (44)	–	
** c/p Stage III/yIII**	4 (14.8)	3 (17)/1 (6)	–	
** c/p Stage IV/yIV**	13 (48.1)	3 (17)/1 (6)	2 (22)/7 (78)	
Grading (n=18)*				0.582
** G2**	4 (22.2)	4 (22)	0 (0)	
** G3**	14 (77.8)	13 (72)	1 (11)	
**Pretherapeutic systemic therapy, n (%)**	9 (33)	2 (11)	7 (78)	**<0.001**
**Patients with tumor resection (n=16)**	16 (59)	15 (83)	1 (11)	**<0.001**
**pT/ypT**				0.887
** pT1**	3 (19)	3 (17)	–	
** pT2**	2 (13)	2 (11)	–	
** pT3/ypT3**	8 (50)/2 (13)	8 (44)/1 (6)	-/1 (11)	
** pT4**	1 (6)	1 (6)	–	
**pN/ypN**				0.309
** pN0/ypN0**	4 (25)/1 (6)	3 (17)/1 (6)	-/1 (11)	
** pN1**	8 (50)	8 (44)	–	
** pN2**	3 (19)	3 (17)	–	
**Pn +**	12 (75)	11 (61)	1 (11)	0.551
**Residual tumor classification**				**<0.001**
** R0**	14 (87.5)	14 (78)	0 (0)	
** R1**	1 (6)	1 (6)	0 (0)	
** Rx**	1 (6)	0 (0)	1 (11)	

pT, pathological T category; UICC, Union for International Cancer Control; pN, lymph node category; Pn, perineural invasion; M, distant metastasis; c, clinical; p, pathological; y, neoadjuvant treatment, * missing data. All data according to TNM classification of 2017. Bold: significant values.

In summary the PDAC (PER+) group shows more characteristics of illness than the other groups.

### Exo-ROR1 in PF discriminates between non-cancer, PDAC (PER-) and PDAC (PER+)

**Figure 2:**
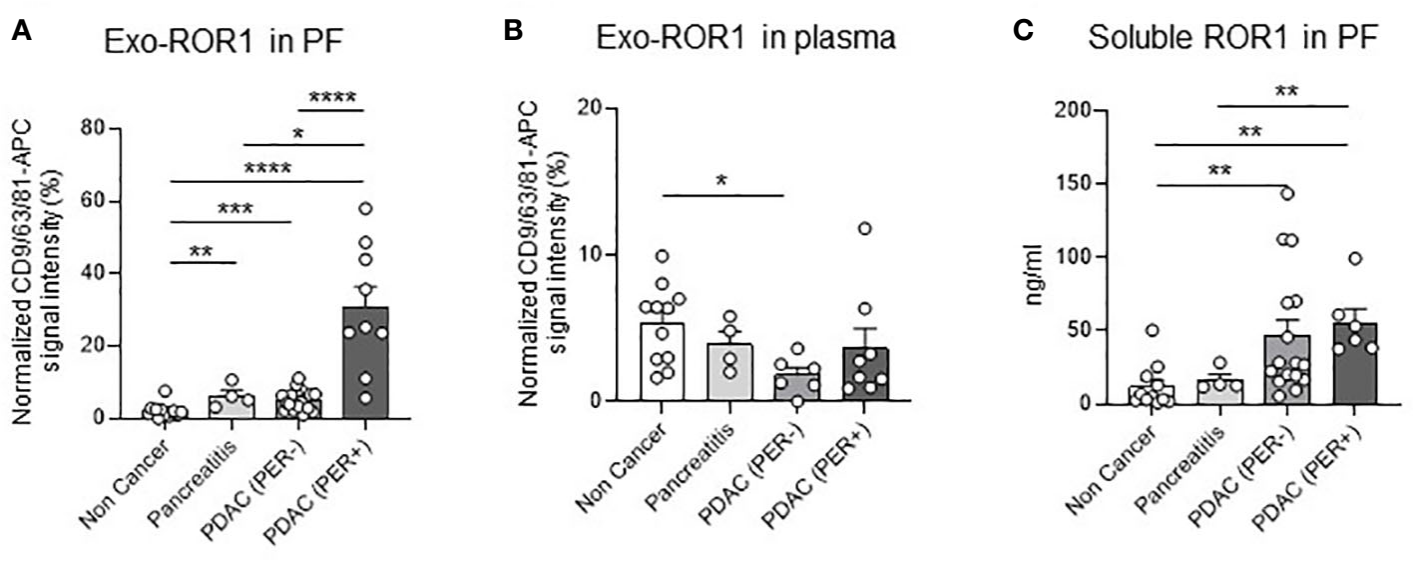
Exo-ROR1 in peritoneal fluid (PF) and plasma and soluble ROR1 in PF. **(A)** Values of normalized CD9/63/81-APC signal intensity of exo-ROR1 (%) in PF. Non Cancer: n=15, Pancreatitis: n=4, PDAC (PER-): n=18, PDAC (PER+): n=9. *p<0.05, **p<0.01, ***p<0.001, ****p<0.0001. **(B)** Values of normalized CD9/63/81-APC signal intensity of exo-ROR1 (%) in plasma. Non Cancer: n=11, Pancreatitis: n=4, PDAC (PER-): n=6, PDAC (PER+): n=8. *p<0.05. **(C)** ELISA: Concentrations of soluble ROR1 (ng/ml) in PF. Non Cancer: n=11, Pancreatitis: n=4, PDAC (PER-): n=16, PDAC (PER+): n=6. **p<0.01.

As a primary result of our study exo-ROR1 in the peritoneal fluid (PF) is able to differentiate between NC, PDAC (PER-) and PDAC (PER+) patients (**Figure 2A**). PDAC (PER+) patients have the highest exo-ROR1 values in PF and can be discriminated from NC (p<0.0001) and PDAC (PER-) (p<0.0001). Likewise, PDAC (PER-) can be discriminated from NC (p = 0.0003). Chronic pancreatitis patients (CP) can be differentiated from NC (p = 0.0036) and PDAC (PER+) (p = 0.0112). There are no differences between CP and PDAC (PER-). The values of the 3 patients with a small liver metastasis did not differ from the other values in the PDAC (PER-) group (**Supplementary Figure S1**) and were therefore included in this group.

In order to see if exosome isolation is necessary, we also performed an ELISA for the detection of soluble ROR1 in PF. The detection of soluble ROR1 in PF allows to distinguish NC from cancer patients (NC vs. PDAC (PER-), p = 0.0012; NC vs. PDAC (PER+), p = 0.0011), but not to differentiate PDAC (PER-) from PDAC (PER+). CP can be separated from PDAC (PER+) (p = 0.0095), but not from PDAC (PER-) (**Figure 2C**).

In plasma exo-ROR1 is not able to distinguish between the groups except that PDAC (PER-) has lower exo-ROR1 values than NC (p = 0.0103) (**Figure 2B**).

**Figure 3:**
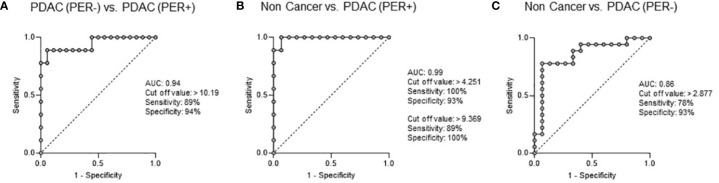
ROC Curve Analysis: Exo-ROR1 in PF. **(A)** PDAC (PER-) vs. PDAC (PER+). AUC: 0.94, cut off value: >10.19, sensitivity: 89%, specificity: 94%. PDAC (PER-): n=18, PDAC (PER+): n=9. **(B)** Non Cancer vs. PDAC (PER+). AUC: 0.99, cut off value: >4.251, sensitivity: 100%, specificity: 93%; cut off value: >9.369, sensitivity: 89%, specificity: 100%. Non Cancer: n=15, PDAC (PER+): n=9. **(C)** Non Cancer vs. PDAC (PER-). AUC: 0.86, cut off value: >2.877, sensitivity: 78%, specificity: 93%. Non Cancer: n=15, PDAC (PER-): n=18.

To evaluate the power of exo-ROR1 as a biomarker we performed ROC-curve analysis, which shows that with a cut-off value of >10.19% of normalized APC-signal intensity PDAC (PER-) can be differentiated from PDAC (PER+) with an AUC of 0.94, a sensitivity of 89% and specificity of 94% (**Figure 3A**). PDAC (PER+) can even be higher differentiated from NC with an AUC of 0.99 and a cut-off value of >4.251% resulting in a sensitivity of 100% and a specificity of 93% or a cut-off value of >9.369% resulting in a sensitivity of 89% and specificity of 100% (**Figure 3B**). PDAC (PER-) can be discriminated from NC with a cut-off value >2.877% ensuing in an AUC of 0.86, a sensitivity of 78% and a specificity of 93% (**Figure 3C**).

Concerning cytology all patients of the PDAC (PER+) group were negative (**Table 3**). In contrast, with the estimated cut-off value of 10.19% for discriminating PDAC (PER-) from PDAC (PER+) 8 of 9 patients (88.9%) were positive for peritoneal carcinomatosis in the exo-ROR1 analysis.

**Table 3:** PDAC (PER+) patients with exo-ROR1 values and cytology: with the cut-off value of 10.19% 8/9 (88.9%) of the PDAC (PER+) patients are positive for peritoneal carcinomatosis (marked in grey) in the exo-ROR1-group. Whereas none of the performed cytologies were positive.

Patient	exo-ROR1 normalized signal intensitiy (%)	Cytology
P268	25,25626987	Negative
P312	48,61277955	Negative
P179	23,7502633	–
P317	43,98630937	Negative
P321	57,98674303	Negative
P383	23,62864685	Negative
P324	5,669797907	Negative
P455	11,07405	–
P416	35,68951	Negative

### ROR1 expression in pancreatic tissue

**Figure 4:**
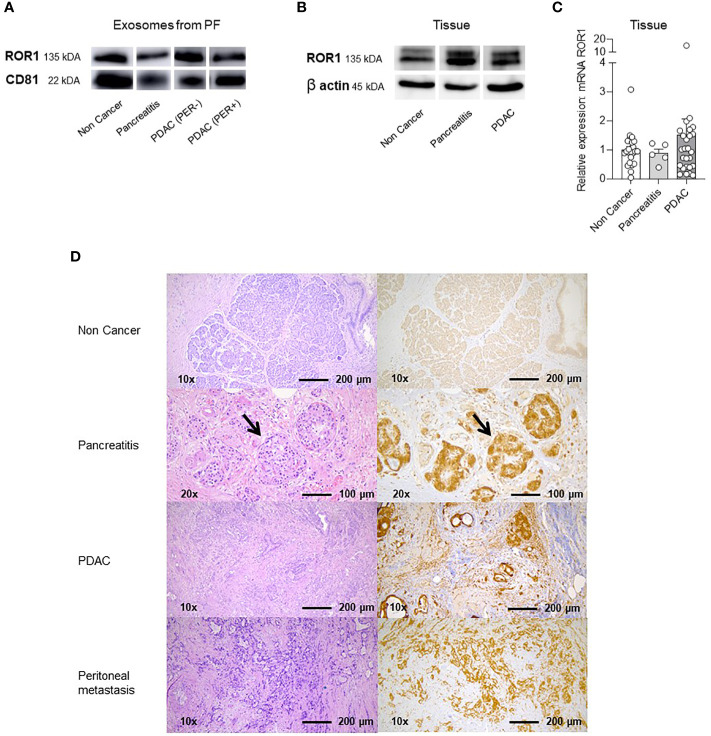
Western Blots (WB), qPCR and Immunhistochemistry (IHC) of exosomes in PF and lysed tissue. **(A)** Western Blot with ROR1 and CD81 of isolated exosomes from PF. Both proteins are expressed on the exosomes of all different groups (Non Cancer, Pancreatitis, PDAC (PER-), PDAC (PER+). For uncropped WB refer to **Supplementary Figure S2**. **(B)** Western Blot of lysed tissue from Non Cancer, Pancreatitis, PDAC. ROR1 and also β-Actin as loading control is expressed in all three groups. For uncropped WB refer to **Supplementary Figure S2**. **(C)** qPCR analysis and relative ROR1 expression of pancreatic tissue. ROR1 is expressed on NC, CP and PDAC tissue showing no significant differences between the groups but a slightly higher expression in the PDAC tissue. **(D)** Immunohistochemistry (IHC) and HE staining of non-cancerous exocrine pancreatic tissue, CP, PDAC and peritoneal metastasis. The exocrine pancreas is ROR1 negative. In the CP tissue ROR1 positive islet cells are shown in the higher magnification (arrows). The fibrotic tissue is negative. In PDAC and the peritoneal metastasis the morphologic tumor cells are ROR1 positive.

To localize the origin of exosomal ROR1 we performed western blots (WB), qPCR, and immunohistochemistry (IHC) from pancreatic tissues. Immunoblot analysis of exosomes revealed expression of ROR1 and CD81 in all groups (**Figure 4A**, **Supplementary Figure S2**), with CD81 being used as loading control for exosomes. Similarly, all pancreatic tissues were positive for ROR1 with β-actin used as loading control (**Figure 4B**, **Supplementary Figure S2**). Additionally, exo-ROR1 was expressed in qPCR in tissue of Non Cancer (n = 24), pancreatitis (n = 5), and PDAC patients (n = 26). There were no significant differences between the three groups even though PDAC patients had a slightly higher relative expression (**Figure 4C**). In IHC, there is no ROR1 expression in the exocrine pancreas. In the pancreatitis sample the islet cells are ROR1 positive, but the fibrotic tissue is negative. Concerning the primary tumor of PDAC as well as in the peritoneal metastasis we see a clear positivity for ROR1 in the morphologic tumor cells (**Figure 4D**).

### High exo-ROR1 expression is associated with lower survival

**Figure 5:**
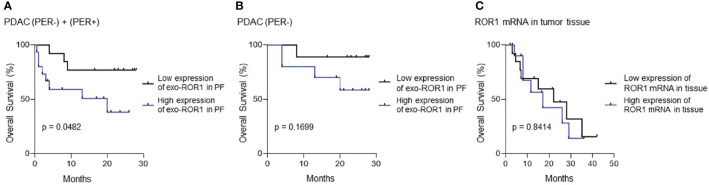
Survival Analysis. **(A)** Survival analysis of all PDAC patients with and without peritoneal carcinomatosis (PDAC (PER+) + PDAC (PER-)). p=0.0482, n=27. **(B)** Survival analysis of only PDAC (PER-) patients without peritoneal carcinomatosis. p=0.1699, n=18. Blue: High expression of exo-ROR1, Black: Low expression of exo-ROR1. **(C)** Survival analysis according to ROR1 mRNA in tumor tissue. p=0.8414, n=25. Blue: High expression of ROR1 mRNA, Black: Low expression of ROR1 mRNA.

To determine the power as a prognostic biomarker we performed survival analysis according to the exo-ROR1 levels. An exo-ROR1 level of >6,62% of normalized APC-signal intensity in PF, which is the median of all exo-ROR1 levels of PDAC (PER+) and (PER-) patients, is associated with a lower overall survival (p = 0.0482) (**Figure 5A**). Hence, patients with a higher exo-ROR1 level in PF die faster. The observation period until data analysis is maximum 28 months for the first included patients. If only including PDAC (PER-) patients (n = 18) with an adapted median for the included values of >4.86% we also see a tendency of lower overall survival of patients with high exo-ROR1 levels, even though not significant (**Figure 5B**). Analysis of ROR1 mRNA expression in tumor tissue of PDAC patients did not reveal differences in overall survival (**Figure 5C**).

### Verification of exosome isolation

**Figure 6:**
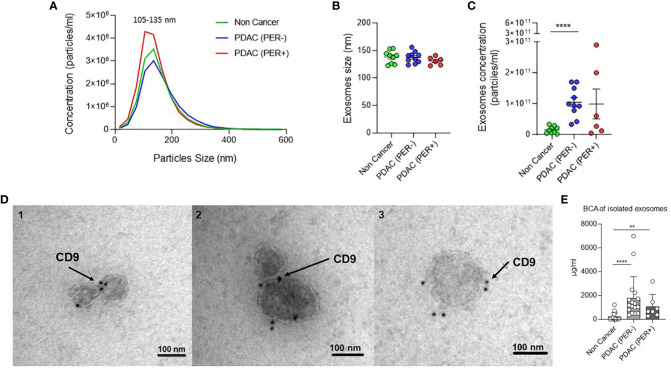
Verification of exosome isolation. **(A)** Nanotracking Analysis (NTA): Exosome concentration and size distribution. Green: Non Cancer n=8, Blue: PDAC (PER-) n=9, Red: PDAC (PER+) n=6. **(B)** Nanotracking Analysis (NTA): Exosomes size distribution by ZetaView analysis showing the mode size of exosomes in 1 ml PF from Non Cancer, PDAC (PER-), and PDAC (PER+) patients. Green: Non Cancer n=8, Blue: PDAC (PER-) n=9, Red: PDAC (PER+) n=6. **(C)** Nanotracking Analysis (NTA): Exosome concentration by ZetaView analysis showing the number of exosomes per milliliter of PF derived from Non Cancer, PDAC (PER-), and PDAC (PER+) patients. ****p<0.0001. Green: Non Cancer n=8, Blue: PDAC (PER-) n=9, Red: PDAC (PER+) n=6. **(D)** Transmission Electron Microscopy (TEM) of isolated exosomes from three PF samples: 1=Non Cancer, 2=PDAC (PER-), 3=PDAC (PER+). Black dots: CD9-immunogold. **(E)** BCA protein concentration analysis of isolated exosomes (µg/ml): Non Cancer: n=15, PDAC (PER-): n=17, PDAC (PER+) n=7. **p<0.01.

To be sure that we isolated exosomes we performed four verification tests: BCA protein assay, Nanotracking analysis (NTA), transmission electron microscopy (TEM), and western blots. The exosome concentration and size distribution in NTA analysis shows a concentration peak of 105–135 nm (**Figure 6A**). No differences in exosomes size distribution could be observed between the three groups (**Figure 6B**). Non-cancerous patients have less concentrations of exosomes compared to PDAC (PER-) patients (p<0.0001) (**Figure 6C**). In BCA assay protein concentrations could be measured in all three groups. Protein concentration is significantly higher in the cancer groups compared to NC (NC vs. PDAC (PER-) p <0.0001; NC vs. PDAC (PER+) p = 0.0028) (**Figure 6E**). CD9-labeled exosomes are visible under the electron microscope (**Figure 6D**). Immunoblot analysis of exosomes revealed expression of ROR1 and CD81 in all groups (**Figure 4A**) as already described above.

## Discussion

With exosomal ROR1 in peritoneal fluid (PF) we discovered a biomarker possibly discriminating between non-cancerous patients, patients with localized, locally advanced, or oligo metastasized PDAC (PER-) and patients with peritoneal disseminated PDAC (PER+). As we do not see the same results in plasma it might be an advantage of the lavage fluid/ascites to be closer to the tumor. PF has been used for staging and analyses in cancer patients before, mostly for cytology (6, 22, 23). Suenaga et al. presented peritoneal lavage tumor DNA as a novel biomarker for predicting peritoneal recurrence in PDAC and compared their results to cytology. The peritoneal tumor DNA biomarker had a much higher sensitivity for peritoneal recurrence than cytology, but lower specificity (23). Cytology in PF of PDAC patients is discussed since positive cytology is prognostically equivalent to metastatic disease (6). However, positive cytology status is not clinically equivalent to gross peritoneal metastasis in PDAC patients. Therefore, curative resection is still recommended regardless of cytology status (21). We did not perform cytology in our peritoneal lavage samples but in 7 of our ascites samples of the PDAC (PER+) group. None of these samples were positive despite gross peritoneal metastasis. Consequently, and since peritoneal lavage for staging in PDAC patients is not common in Germany we did not focus on cytology.

Furthermore, exo-ROR1 in PF can also be used as a prognostic marker since overall survival with high expression of exo-ROR1 was significantly lower than with low expression. Zhang et al. also presented that ROR1 expression on tumor tissue correlated with poor clinical outcome in human ovarian cancer (11). Same results were found in lung adenocarcinoma (12). In contrast, Liu et al. (13) show that high ROR1 mRNA expression in PDAC correlate with a favorable overall survival. In our cohort we could not show any significant differences regarding survival between high and low mRNA expression in tumor tissue. Considering that we have less samples and used qPCR instead of next generation sequencing the function of ROR1 has to be further explored to understand the different expressions in different samples. However, in IHC ROR1 expression is higher in PDAC and also in peritoneal metastases compared to normal exocrine pancreatic tissue. Therefore, ROR1 might play a role in tumor progression.

Nevertheless, exo-ROR1 in PF might shed light on future diagnostic and therapeutic concepts in PDAC. In this regard the receptor tyrosine kinase-like orphan receptor 1 (ROR1) seems to be a promising protein. ROR1 is detectable in embryonic tissue, mostly absent in adult tissue and overexpressed in many types of malignant tumors (9, 10, 24). These characteristics qualify ROR1 as a biomarker and assumably ideal drug target for cancer therapy. Yamazaki et al. showed the importance of ROR1 in promoting tumor-initiating cells and hyperproliferation in PDAC. They demonstrated that ROR1 depletion suppresses tumor growth, recurrence after chemotherapy, and metastasis in PDAC (15). This highlights the therapeutic feasibility of ROR1. To date, several therapeutic strategies against ROR1 have been developed (10). Cirmtuzumab, a monoclonal antibody targeting ROR1, is evaluated in clinical trials regarding chronic lymphocytic leukemia, mantle cell lymphoma and breast cancer (10). Based on ROR1-targeted monoclonal antibodies other therapeutic strategies such as antibody drug conjugate, bispecific T cell engager (BiTE), and chimeric antigen receptor (CAR) T cells have also been developed and are evaluated in clinical trials (10). According to our results a concept of intraperitoneal chemotherapy with a ROR1 targeted therapy could be an interesting approach in the future.

Although we detected significantly different results in the ELISA of soluble ROR1 in PF, soluble ROR1 was not able to differentiate between PDAC (PER-) and PDAC (PER+). Therefore, the isolation of exosomes seems to be a useful tool to get more precise and discerning results.

To investigate the origin of the ROR1 positive exosomes tissue analysis was performed. In Western blot analysis ROR1 is expressed in non-cancerous pancreatic tissue (NC) as well as chronic pancreatitis (CP) and PDAC. qPCR also reveals ROR1 expression in NC, CP as well as PDAC tissues. The positive results in NC and CP might result from the islet cells, which are known to be positive (9) and can also be shown in the IHC. In IHC, we see a shift to ROR1 positive cells from normal exocrine tissue to the primary tumor as well as the peritoneal metastasis. Likewise, Liu et al. (13) and Yamakazi et al. (15) proved the expression of ROR1 in PDAC. ROR1 seems to play an important role in metastasizing of PDAC since Yamakazi et al. found that ROR1high cells are abundant in metastatic lesions of PDAC patients, suggesting that these ROR1high cells were the origin of metastases (15). Therefore, some transformation must occur during tumor growth and expansion, suggesting that ROR1 positive exosomes in PF might play an important role in the development of peritoneal carcinomatosis in PDAC patients. This needs to be elucidated in further studies.

For sampling of PF surgery with the possibility of perioperative complications must be performed. Most patients with localized PDAC receive primary surgery anyways. For borderline tumors pretherapeutic explorative laparoscopy could be performed to exclude peritoneal carcinomatosis and to obtain the PF similarly to gastric cancer patients (6). Peritoneal lavage can be particularly useful in a patient population with no clinical evidence of metastatic disease and radiographically occult peritoneal carcinomatosis in order to reduce the occurrence of unnecessary laparotomy and non-curative operative resections. Maybe these patients will profit from neoadjuvant therapy in the future. Even percutaneous peritoneal lavage is described and originated from trauma setting as a diagnostic lavage for rapid diagnosis of intraabdominal injury (25). It can be done in Seldinger technique as well as using a Veress needle and was tested in a prospective randomized trial as similar safe as an open technique (26).

Regarding the baseline characteristics there are some significant differences between the groups which show that the PDAC (PER+) patients are sicker than the other groups. The ASA score differs between the groups which is reasonable due to the severe illness of the cancer patients compared to the non-cancerous patients. Nevertheless, the ASA score is depending on the anesthesiologist who is assigning it to the patients (27). It could be argued that all tumor patients should at least receive an ASA score of 3 since independent of all other comorbidities a pancreatic tumor seems to be one of the most threatening diagnoses. The preoperative blood results also underline the sickness of the PDAC (PER+) patients since they show lower hemoglobin values, higher CRP and ɤGT levels and lower quick values. Higher bilirubin levels in the PDAC groups are also reasonable due to cholestasis if the tumor is located in the pancreatic head.

In the tumor characteristics of the PDAC patients we actually expect differences between the groups since we compare different tumor stages. Worth mentioning is the fact that 78% of the PDAC (PER+) patients received a preoperative systemic therapy compared to 11% in the PDAC (PER-) group. Most of the PDAC (PER+) patients had chemotherapy or radiochemotherapy for known inoperable PDAC. Whereas most PDAC (PER-) patients received primary surgery and only locally advanced PDAC patients received neoadjuvant chemotherapy or even radiochemotherapy. Due to the low number of samples comparison within the groups is not possible. In the PDAC (PER+) group two patients did not receive neoadjuvant systemic therapy. One of these patients has a low exo-ROR1 value, the other a high value. Therefore, exo-ROR1 is not purely driven by systemic therapy. Still, an influence of neoadjuvant therapy on exo-ROR1 in the PF cannot be excluded and should be evaluated in further studies.

In BCA and NTA analysis of the isolated exosomes the protein and particle concentrations differ between the groups. The highest concentrations in PDAC (PER-) in BCA could be explained through a slight overestimation of the concentrations with the dilution factor of 4. Some of the patients might have had more intraabdominal fluid than 30–50 ml due to the cancer or other reasons. Besides that, the protein concentrations of the NC group are striking low, which is also shown in the exosome concentration of the NTA analysis. This might be directly correlated to the cancer in the other groups. There are similar results in blood. The blood of healthy individuals may contain over 2000 trillion exosomes, whereas that of cancer patients contains 4000 trillion exosomes (8). Thus, tumor cells may produce and secrete more exosomes compared to normal cells.

The present study has some limitations. First, due to a lack of samples we did not include as many patients in the plasma and ELISA analysis as in in the exo-ROR1 in PF analysis. Second, there might be a slight overestimation of the concentrations in the BCA and ELISA with the dilution factor of 4. But in order to get an equal representation, we had to define one way of analyzing. Third, we are lacking in sample size of CP patients. But as we wanted to show the differences in cancer patients this can be neglected.

With exo-ROR1 in PF we found a promising diagnostic biomarker possibly discriminating between NC, PDAC (PER-) and PDAC (PER+) and might shed light on future diagnostic and therapeutic concepts in PDAC. Additionally, it might be useful as a prognostic marker since patients with high exo-ROR1 in PF have a lower overall survival. The validity of this marker has to be tested in larger studies.

## Data availability statement

The raw data supporting the conclusions of this article will be made available by the authors, without undue reservation.

## Ethics statement

The studies involving humans were approved by Ethikkommission der Friedrich-Alexander-Universität Erlangen-Nürnberg Krankenhausstraße 12 91054 Erlangen. The studies were conducted in accordance with the local legislation and institutional requirements. The participants provided their written informed consent to participate in this study.

## Author contributions

Conceptualization: AM, DK, and GW. Methodology: AM, DK, AA, AG, J-OB, JD, CG, and GW. Formal analysis: AM, DK, AA, PD, and GW. Investigation: AM and AA. Resources: AM, AA, PD, MP, AB, MB, CK, AJ, AD, KW, DH, TB, DS, YZ, JD, SL, SS, RG, and GW. Data curation: AM, AA, LR, BK, IS, and SM. Writing – original draft preparation: AM and GW. Writing – review and editing: all authors. Visualization: AM, AA, AG, and GW. Supervision: GW. Project administration: GW. Funding acquisition: GW. All authors have read and agreed to the published version of the manuscript. All authors contributed to the article and approved the submitted version.

## Supplementary material

Supplemental Figure S1 Expression of normalized CD9/63/81-APC signal intensity of exo-ROR1 (%) in PF. Non Cancer: n=15, Pancreatitis: n=4, PDAC (PER-): n=15, PDAC (HEP): n=3, PDAC (PER+): n=9. *p<0.05, **p<0.01, ***p<0.001, ****p<0.0001.

Supplemental Figure S2 Uncropped Western blots.
