# Supplementary figures and images for: Exosomal ROR1 in peritoneal fluid identifies peritoneal disseminated PDAC and is associated with poor survival
Source: Front Immunol. 2024 May 23;15:1253072. doi: 10.3389/fimmu.2024.1253072 (PMC11153717; doi:10.3389/fimmu.2024.1253072)

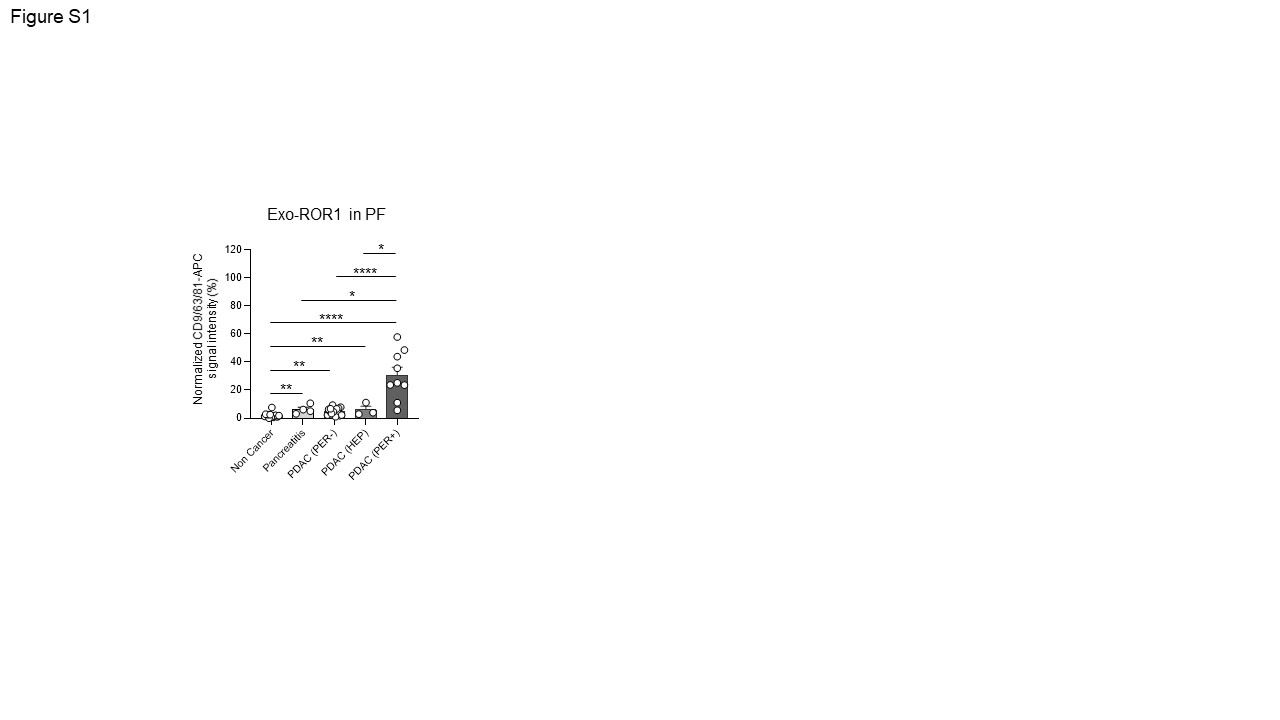

Supplement: Supplemental Figure S1 — Expression of normalized CD9/63/81-APC signal intensity of exo-ROR1 (%) in PF. Non Cancer: n=15, Pancreatitis: n=4, PDAC (PER-): n=15, PDAC (HEP): n=3, PDAC (PER+): n=9. *p<0.05, **p<0.01, ***p<0.001, ****p<0.0001. [file Image_1.jpeg]

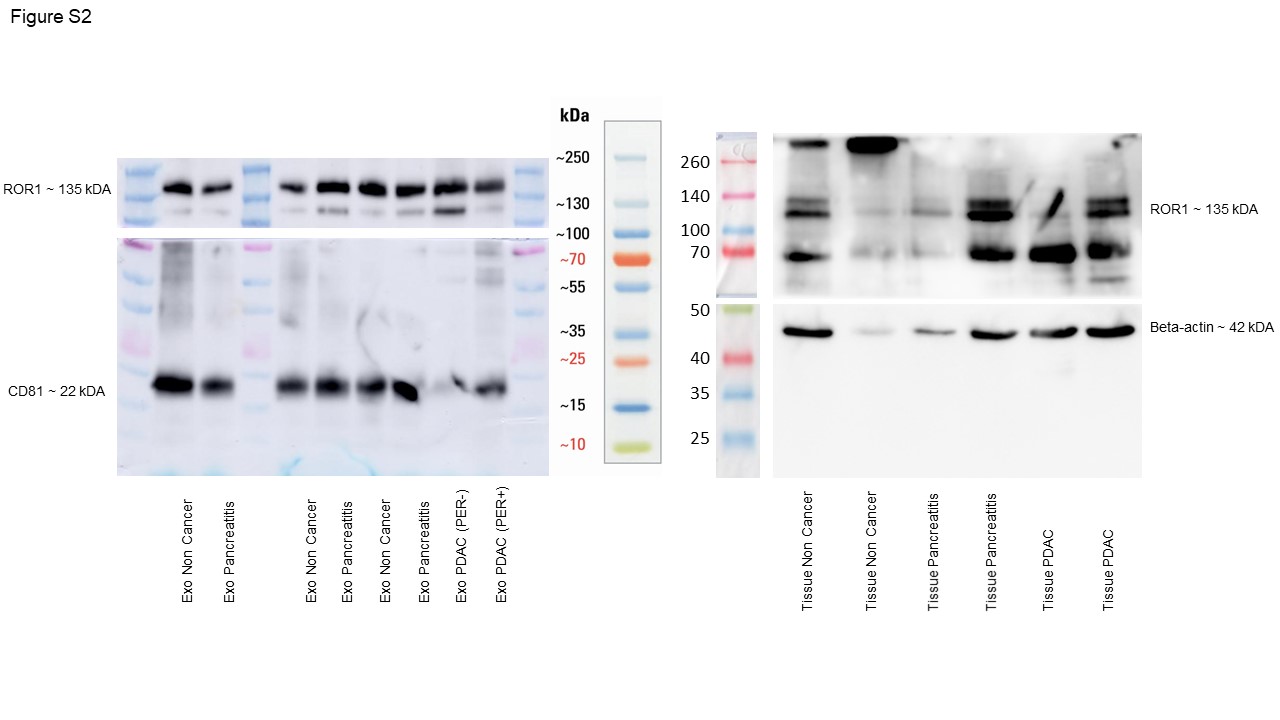

Supplement: Supplemental Figure S2 — Uncropped Western blots. [file Image_2.jpeg]
